# Love and peace across generations: Biobehavioral systems and global partnerships

**DOI:** 10.1016/j.cpnec.2021.100092

**Published:** 2021-10-09

**Authors:** James F. Leckman, Liliana Angelica Ponguta, Gabriela Pavarini, Sascha D. Hein, Michael F. McCarthy, Haifa Staiti, Suna Hanöz-Penney, Joanna Rubinstein, Kyle D. Pruett, M. Yanki Yazgan, N. Shemrah Fallon, Franz J. Hartl, Margalit Ziv, Rima Salah, Pia Rebello Britto, Siobhán Fitzpatrick, Catherine Panter-Brick

**Affiliations:** a Child Study Center, Yale University, New Haven, CT, USA; b Departments of Psychiatry, Psychology and Pediatrics, Yale University, New Haven, CT, USA; c Early Childhood Peace Consortium, New York, NY, USA; d Ethox Centre, Wellcome Centre for Ethics and Humanities, Nuffield Department of Population Health, University of Oxford, Oxford, UK; e Freie Universität, Berlin, Germany; f Warner School of Education and Human Development, University of Rochester, Rochester, NY, USA; g Empathy for Peace, Toronto, Canada; h Mother Child Education Foundation (AÇEV), Istanbul, Turkey; i United Nations Sustainable Development Solutions Network, Paris, France; j Güzel Günler Clinic, Istanbul, Turkey; k University Web Operations, Yale University, New Haven, CT, USA; l The International Networking Group on Peacebuilding with Young Children, Belfast, Northern Ireland, UK; m Country Representative, UNICEF, Lao’s People Democratic Republic (Lao PDR); n The Early Years – the Organization for Young Children, Belfast, Northern Ireland, UK; o Jackson Institute for Global Affairs and Department of Anthropology, Yale University, New Haven, CT, USA

**Keywords:** Early childhood development, Neurobiology, Parenting, Peacebuilding, Policy, Sustainable development goals

## Abstract

Children's environments - especially relationships with caregivers - sculpt not only developing brains but also multiple bio-behavioral systems that influence long-term cognitive and socioemotional outcomes, including the ability to empathize with others and interact in prosocial and peaceful ways. This speaks to the importance of investing resources in effective and timely programs that work to enhance early childhood development (ECD) and, by extension, reach communities at-scale. Given the limited resources currently devoted to ECD services, and the devastating impact of COVID-19 on children and communities, there is a clear need to spur government leaders and policymakers to further invest in ECD and related issues including gender and racial equity. This essay offers concrete examples of scholarly paradigms and leadership efforts that focus on child development to build a peaceful, equitable, just, and sustainable world. As scholars and practitioners, we need to continue to design, implement, assess, and revise high-quality child development programs that generate much-needed evidence for policy and programmatic changes. We must also invest in global partnerships to foster the next generation of scholars, practitioners, and advocates dedicated to advance our understanding of the bio-behavioral systems that underlie love, sociality, and peace across generations. Especially where supported by structural interventions, ECD programs can help create more peaceful, just, and socially equitable societies.

## Highlights

• Science has documented the lasting long-term benefits of Early Childhood Development programs from pre-gestation onwards.
• Sadly, many children and their families, especially in conflict-affected areas, are exposed to toxic environments.
• In addition, too many children are now being exposed violence online and, in their homes, schools, and communities.
• There is a need for government leaders and policymakers to invest in ECD programs that promote gender and racial equity.
• We also need to partner with children, youth, and their families to build more a peaceful and socially cohesive world.

*Difference is an accident of birth and therefore should never be a source of hatred or conflict. The answer to difference is to respect it. Therein lies a most fundamental principle of peace – respect for diversity.*

John Hume

*Peace is integral to human existence. In everything we do, in everything we say, in every thought we have, there is a place for peace. Early childhood affords a window of opportunity to instill what each individual needs to become an agent for peace and nonviolence.*

H.E. Ambassador Anwarul K. Chowdhury

## 1 Introduction

The case for the importance of early childhood development (ECD) has been made. To-date, scholars and practitioners from across the biological and social sciences - including anthropology, economics, education, global health, neurobiology, pediatrics, psychology, and social work - have clearly documented the impacts of early life events and social relationships for developmental trajectories and later-life neuropsychosocial health. Specifically, early childhood experiences set the stage for the emergence of neurodevelopmental and neuropsychiatric wellbeing and/or disorders [1], for they impact the bio-behavioral systems that underlie our motor, cognitive, and socio-emotional development [[2], [3], [4]]. The degree to which children are able to reach their full developmental and economic potential rests in part on the interaction between genes and environments, or “nature *via* nurture” [2,5,6]. These findings convey a message of hope and change. It is increasingly apparent that the early years of life set the foundations for a cascade of related outcomes - not only for the wellbeing of individual children, but also for the societies in which they live [7,8]. However, despite compelling evidence of significant benefits, especially in conflict or resource-poor contexts, high quality ECD programs have rarely been fully prioritized by governments or policymakers [9].

This paper reviews the interdisciplinary body of knowledge that can help us move ahead as a global community to build a peaceful, equitable, and sustainable world. We describe a series of successful ECD programs in conflict-affected settings, emphasizing the importance of cross-sectoral partnerships to drive social change through effective ECD programming. The paper is aimed at researchers interested in ECD, peacebuilding, and cross-sectoral collaborations, as well as policymakers and professionals working in nonprofits or development. We address the bio-behavioral systems underlying love, attachment, social cohesion, and peace, and the importance of nurturing care for sustaining peace at personal, family, and societal levels. We then review the leadership efforts of nongovernmental organizations such as the Early Childhood Peace Consortium (ECPC) and the Early Childhood Development Action Network (ECDAN), which seek to inform policymakers and encourage them to improve existing ECD systems. We reflect on partnerships and the kind of leadership necessary for taking concrete steps to build peace and empathy in the world. Such efforts could not be timelier as we grapple with the challenges of a global pandemic, persistent inequalities, ongoing violence, unjust policies, and contested state leadership.

It is important not to restrict the scope of parenting programs by focusing solely on mother-child dyads. Fathers matter for child and family wellbeing, cognitive development, and peacebuilding. A recent expansion of research based on parent-child interactions has focused on programs, which reduce child abuse, neglect, harsh discipline, and community conflict through supporting father engagement in caregiving [10]. There is strong, global evidence for engaging fathering figures in the lives of young children to reduce harsh discipline, parental stress and depression, and interpersonal violence in the home; there is also emerging evidence that engaging fathers and grandparents in nurturing care will impact child development outcomes and can help abate conflict within neighborhoods and family networks [[11], [12], [13], [14]]. Consequently, policies and programs can be encouraged to widen interventions to actively recruit and maintain the involvement of fathers and other caregivers to foster children's development. There is also growing recognition of the diversity of family units, including LGBTQ parents, single mothers by choice, and co-parents. This reality calls for more inclusive community ECD programming [15,16].

## 2 Biobehavioral systems of love, attachment, social cohesion, and peace

The bio-behavioral systems that underlie the development of selective social bonds between children, families, and communities are deeply rooted in mammalian evolution [114]. There is an ever-expanding scientific literature concerning the impact of child-rearing environments, both positive and negative, on the functioning of human bodies and brains. However, many questions remain concerning the interplay of neurobiology, physiology, cognitive and social development, especially for children and adolescents living with stress, insecurity, or poverty, and how biobehavioral systems respond to targeted interventions [[17], [18], [19]]. Recent work has broadened our perspective on the “environome” to include physical and cultural influences, and to expand gene-by-environment studies through comparative work across a range of contexts [6,20]. It is now clear that early life experiences contribute to the functional capacity of the brain in ways that directly affect an individual's capacity for empathy and prosocial behavior. For instance, many individuals show protective responses to stress and trauma [97,98]. Factors that influence resilience pathways include social support, the timing or intensity of adversity exposures, and epigenetic mechanisms, which are key players with regard to how adverse and traumatic events can become biologically embedded [99]. Thus, programs that effectively boost responsive parenting and psychosocial resilience may have a range of beneficial impacts, potentially changing the signatures of toxic stress and adverse childhood experiences, the functioning of cognitive, hormonal and immune systems, and the plasticity of DNA transcription from one generation to the next [21,22,98,115,116].

Early childhood is a “sensitive period” of development that has foundational effects on a broad range of skills and abilities [23]. Infants and young children grow faster and learn more in their early years than in any other period of life. In the first year of life, more than 1 million new neural connections per second are made, a pace that is *never* achieved again [24]. By 3 years of age, a child's brain is *twice as active* as an adult brain. As we discuss below, optimal child development is dependent on children receiving “nurturing care” so that they grow up in safe environments with adequate nutrition, responsive caregiving, opportunities for early learning, and access to good health care [2].

For example, the neuropeptides oxytocin (OT) and vasopressin (VPN) and their receptors play a key role in the formation of social bonds. The OT-VPN system interacts with the hypothalamic-pituitary-adrenal stress response axis as well as with a number of other pathways that are key to human reproduction and survival [25]. Importantly, the OT-VPN system is a fundamental element in the formation of maternal and paternal-child attachment and interpersonal bonds as well as being intimately involved in sexual behavior, labor and delivery, and breastfeeding [25,26]. The OT-VPN system influences, and is influenced by social interactions including trust, cooperation, empathy, and peace. Although VPN is primarily produced in the hypothalamus, OT is produced throughout the body including the heart, thymus, gastrointestinal tract, and reproductive organs, in addition to the hypothalamus. The integrated OT-VPN system is part of, and interacts with, many other elements of our sensory, reward, and reproductive pathways in the body and the brain. This system is also intimately involved not only with the formation and maintenance of social bonds but also with an individual's response to stress and adversity [[27], [28], [29]]. In addition to the findings from animal model systems, initial human studies have documented another important role of the OT-VPN system. High levels of peripheral OT have been associated with loving, synchronous, social interactions between responsive caregiving by mothers and stimulatory play by fathers [[30], [31], [32], [33]]. Intranasal OT has been shown to modulate anxiety, responsiveness, trust, and altruism. For example, more securely attached individuals as adults show an increased reward response to being with their child and less of an aversive reaction to hearing the baby's cries [34].

The social neuroscience literature has identified multiple distinct, but interrelated and malleable, neural systems involved in parenting for both mothers [[35], [36], [37]] and fathers [38]. These circuits include the reward and stress-response pathways as well as the mirroring system, the mentalizing system, and the salience network. Allostasis, has also been identified as an important brain-centered factor involved in physiological regulation [100]. One of the most detailed explorations of the impact of a successful mother-child intervention on the neural circuitry of the mother's brain has come from a longitudinal study in a parenting program that promoted intersubjectivity in low-income urban communities in Michigan, U.S.A [36,39]. Impacts on neural circuits involved the interconnections of the dorsomedial prefrontal cortex, the superior temporal gyrus, the prefrontal cortex, the amygdala, the nucleus accumbens and the periaqueductal gray have been documented [36]. Many of these same circuits are strengthened by interventions designed to enhance compassion towards fellow humans [40].

As mentioned above, another emerging research area relevant to social cohesion and peacebuilding is the field of epigenetics. Epigenetic mechanisms involve changes in the regulation of gene activity and expression through alterations of the DNA structure and chromatin function that are not dependent on the DNA sequence that was inherited from a parent [41,42]. For example, animal studies have demonstrated that future parental behaviors can be shaped by epigenetic modifications and can have a lasting influence on patterns of gene expression in the developing brain, starting with fetal development. Investigators at McGill University, Canada, have documented that high versus low levels of licking and grooming behaviors by mother Norwegian rats during the first few days of postnatal life are associated with epigenetic modifications of the regulatory regions of a number of genes that can produce enduring effects on gene expression, leading to individual differences in stress response and future maternal behavior by their daughter pups [43]. In addition to the epigenetic research done with rodents, the results of which are not directly applicable to humans, a growing number of human studies provide evidence that experience-dependent epigenetic alterations impact the development of the brain more than that of any other organ [44]. There are even experience-driven epigenetic changes, associated with the nature of maternal care and stress exposure, that are heritable and can impact brain function across generations [21]. As with other markers of the bio-behavioral systems reviewed above, the care of mothers, fathers, and other caregivers are important contributors to neuroplasticity and epigenetic changes.

## 3 The importance of nurturing care

The most fundamental and formative experiences in the early years of life come from the extent to which young children receive nurturing care from their family and community. Nurturing care refers to five interrelated components: safety, adequate nutrition, access to health services, responsive caregiving, and early learning opportunities. This is manifested through behaviors and knowledge about maintaining the safety of the children (e.g., routines, protection from violence, abuse, neglect, harm, and environmental pollution) and access to health services (e.g., health, hygiene care, and feeding). Responsive caregiving includes stimulation (e.g., talking, singing, and playing); responsiveness (e.g., early bonding, secure attachment, trust and sensitive communication). Each of these components is constrained by structural conditions affecting the life chances and life choices of a given family [2]. Nurturing care can be supported by a range of interventions delivered pre-pregnancy, during gestation, the newborn period, infancy, and early childhood [2]. Many of these interventions have shown to have lifelong benefits including improved health and wellbeing, and increased ability to learn and earn, as well as reductions in morbidity, mortality, disability, and injury [2]. Such evidence is pivotal to the endorsement of ECD as a transformative element in the 2030 Sustainable Development Goals (SDGs), a collection of 17 global goals designed to be a “blueprint to achieve a better and more sustainable future for all” [45]. The SDGs were approved in 2015 by the United Nations General Assembly [46]. To achieve the SDGs by 2030, it is clear that national policies and programs for all children need to be strengthened. Indeed, the recent WHO-UNICEF-Lancet Commission report prepared by the World Health Organization and UNICEF – entitled “A future for the world's children?” - makes a compelling case that the needs of children should be at the center of SDG policies [7,47]. As stated on July 18, 2020 by H E. António Guterres, the ninth Secretary-General of the United Nations, on the occasion of Nelson Mandela's Day:

“*Governments must prioritize equal access, from early learning to lifelong education. Neuroscience tells us that pre-school education changes the lives of individuals and brings enormous benefits to communities and societies*."

Unfortunately, many children in our world do not receive adequate nurturing care [7]. Multiple factors such as socioeconomic stresses underpin the wellbeing and mental health of caregivers, which may increase the risk of adequate care not being provided to children [23,48]. Specifically, improvements in caregiver mental health and parenting-related stress can be expected to have demonstrable impacts on children's developmental outcomes [49], especially in conflict and refugee settings given how caregiver-child mental health cascades across generations [50,51] and in settings of food insecurity where parental depression and anxiety can lead to child behavioral problems [52]. Black et al. (2017) estimated, based on proxy measures of stunting and poverty, that more than 250 million, or 43% of children under the age of 5 years of age are at risk of not achieving their developmental potential [53]. Because of these observations, it is pivotal that researchers and the global community of scholars and practitioners seek to better understand family and community dynamics and their implications for child development. Such knowledge is crucial to better tailor evidence-based ECD services to family and community needs, and, consequently, promote the long-term acceptability, uptake, and sustainability of programs. In this respect, there is an urgent need to address the inequities linked to gender and racial discrimination – these impact human health and development, early in life, and have inter-generational consequences [119].

One aspect of this reality is the mass incarceration of individuals from minority and disadvantaged backgrounds, which can have an adverse impact on children. This important issue now is the call for research and structural change [[101], [102], [103], [104], [105], [106], [107], [108], [109], [110]]. Many political leaders are now actively considering how best to address and counteract mass incarceration and systemic racism in COVID-19 times [54; <https://www.whitehouse.gov/briefing-room/presidential-actions/2021/03/08/executive-order-on-establishment-of-the-white-house-gender-policy-council/) and Advancing Racial Equity (see: https://www.whitehouse.gov/briefing-room/presidential-actions/2021/01/20/executive-order-advancing-racial-equity-and-support-for-underserved-communities-through-the-federal-government/). There are also calls for solidarity and social justice to bring about a fairer immigration policy towards migrant families at the United States-Mexico border [55,56].

Many other challenges to optimal child development point to the need for urgent global action. Every year, hundreds of millions of children are exposed to domestic violence and harsh parenting, often from an early age [57]. It is estimated that globally, one out of two children aged 2–17 years old suffer some form of violence each year [58] and one in ten children are sexually abused before the age of 18 years (https://www.childhood-usa.org/). Other challenges include the presence of parental psychopathology and the use of addictive substances by parents. As a result, hundreds of millions of children fail to reach their full developmental potential [23]. Another core issue to the field of child rights and protection is regulating internet use given the proliferation of digital devices and electronic games with graphic violent content, with potential consequences on brain development, behavior, and risks of child abuse or exploitation online (for example, see the United Nations Broadband Commission for Sustainable Development “Child Online Safety Report” [59]).

## 4 Early Childhood Peace Consortium (ECPC) and early childhood action network (ECDAN)

We turn to leadership efforts – such as those of ECPC and ECDAN - that focus on comprehensive child development and global partnership initiatives to build a more peaceful, equitable, and sustainable world. The ECPC was founded in the belief that children and families can be agents of change to promote love, empathy, and peace [60]. Its formation, in 2013, was inspired by a fusion of high-level advocacy and interdisciplinary science with the ongoing support of UNICEF and several NGOs including the Fetzer Institute (https://fetzer.org/community/bboisture). Thoughts too of the landmark resolution at the United Nations endorsed a ‘culture of peace’ [61] – under the leadership of H.E. Ambassador Anwarul K. Chowdhury, who said that “*early childhood affords [..] opportunity to become an agent for peace and nonviolence*”. In addition, the scientific and programmatic evidence in support of ECD programs as a pathway to peace was thoroughly reviewed by a large group of interdisciplinary experts during the meetings, such as the 15th Ernst Strüngmann Forum [8,62].

The mission of the ECPC is to create an inclusive movement for peace, social cohesion, and social justice. Its three main goals, presented in Table 1, are well aligned with the goals of the 2030 “Agenda for Sustainable Development” and the “Sustaining Peace Resolutions”. The ECPC is currently actively engaged in supporting youth leaders to help achieve such goals (see section 7 below). Up-to-date information concerning ECD programs is now being disseminated digitally via the ECPC website; this includes, for example, the “ECPC Global Call to Action in response to COVID-19 for children in fragile and conflict-affected settings: The promise of early childhood development” (see: https://ecdpeace.org/work-content/read-ecpc-global-call-action-reponse-covid-19). In leveraging early childhood development as a pathway to empathy and sociality, ECPC team members have worked towards four dimensions of peace: 1) as an outcome defined by the cessation of violence, 2) a process to build non-violent social bonds, 3) a disposition predicated on prosocial actions and respect for human dignity, and 4) a culture that fosters a sense of global citizenship.

**Table 1:** Goals of the early childhood peace consortium <https://ecdpeace.org/>

**Goal 1.** To link emerging knowledge from bio-behavioral and environmental sciences with existing evidence to increase investment, advocate for and create local and sustainable programs, as well as policies and systems for peacebuilding and reduction of violence through high quality ECD programs.
**Goal 2.** To contribute to peacebuilding by focusing on ECD and engaging families, communities, civil society, governments and philanthropists through science, practice and policy.
**Goal 3.** To disseminate information to practitioners, scholars, and policymakers, to build a global movement that values young children and families as agents for peace.

The ECDAN was launched in 2016 by the World Bank and UNICEF and more than 80 organizations across the globe, in an effort to catalyze collective action on behalf of young children and their families, facilitating knowledge exchange and coordinating advocacy for investing in quality services (see: https://www.ecdan.org/). ECDAN works in close alignment with seven regional networks, each moving ahead to create, test, refine, and bring to scale high-quality ECD services [14,[63], [64], [65]]. Together, ECDAN and the regional networks are creating a movement to engage diverse partners in the field of ECD (see: https://bernardvanleer.org/ecm-article/2018/supporting-refugee-internally-displaced-and-marginalized-host-community-parents-in-the-arab-region/). Interventions will continue to improve with the growth of developmental science, but in most instances, ECD programs must build on existing delivery platforms to enhance their feasibility and ensure that they can be successfully scaled-up [[63], [64], [65]]. Global efforts to identify promising programs and providers are now increasingly available, thanks in part to ECDAN's global network that is dedicated to expanding the impact of ECD programs and policies by connecting stakeholders across regions and sectors to take collective action (see: https://www.ecdan.org/index.html).

There are also multiple examples of local initiatives and organizations that support young people's involvement in peacebuilding such as the Africa Youth for Peace and Development (https://africayouth.webs.com) and the Sou da Paz Institute in Brazil (https://soudapaz.org). Another example is the Arab-German Young Academy of Sciences and Humanities (AGYA; https://agya.info/). AGYA was established as a collaborative effort between researchers in 22 countries in the Arab world and Germany. AGYA's initiatives focus specifically on education at the interface of science and society. In AGYA, early-career researchers seek to jointly address the challenge of access to quality ECD services by building on mutual trust and knowledge exchange.

In brief, the evidence across sectors, populations, and settings for the importance of ECD programs continues to expand. It is clear that in order for ECD interventions to be successful, strategic, equitable, and sustainable, they need to be implemented as multi-sectoral intervention packages, firmly anchored in nurturing care.

## 5 Exemplars of ECD programs in conflict-affected regions

Examples of ongoing programs include those implemented in conflict-affected regions, funded by the National Institute for Health Research (NIHR) in the United Kingdom through the LINKS initiative (The NIHR Global Health Research Group on Early Childhood Development for Peacebuilding). These include programs in Columbia, Palestine, Egypt, Mali, Kyrgyzstan, Tajikistan, Timor-Leste, and Vietnam [3]. We detail below ECD initiatives in three conflict-affected regions to better draw the connections between parenting, bio-behavioral development, and peacebuilding efforts. While there is growing evidence for a catalyst role of ECD programs and families in conflict-affected regions, it is naive to think that - without larger structural interventions - ECD programs alone will sustain more peaceful, just, and equitable societies [[66], [67], [68], [69]].

**Northern Ireland.** The Good Friday Agreement, led by the late John Hume, has set the stage for ECD programs, focused on peacebuilding in Northern Ireland. The Media Initiative “Too Young to Notice?” was developed following ground-breaking research showing that children as young as 3 years of age were developing negative and prejudicial attitudes to those who were different from them [70]. Several qualitative evaluations and a randomized clinical trial were conducted to assess the impacts of The Respecting Difference Programme implemented in local communities [71,72]. The results demonstrated that this program had strong positive effects on the social and emotional well-being of young children and a promising impact on teachers and parents in terms of their ability to deal with issues of difference and promoting positive aspects of identity. ECD peacebuilding efforts in Northern Ireland are still ongoing, thanks to the efforts of organizations such as the non-governmental organization (NGO) Early Years (see: https://www.early-years.org/).

**Turkey.** As a result of forced displacement in the wake of the Syrian conflict that began over a decade ago, there are an estimated 3.6 million Syrian refugees now living in Turkey. While 1.7 million of those registered are children, 860,000 of them are school-aged and have limited access to schools. The European Union, with additional support of the Turkish Ministry of National Education, has been actively involved in the integration of Syrian children into the Turkish Education System. Its program includes Turkish and Arabic language courses, as well as catch-up education and remedial/support classes, and transportation services for students [73]. In addition, the Mother-Child Education Foundation (AÇEV), an NGO based in Turkey, has been implementing a summer program for preschool children (see: https://www.acev.org/wp-content/uploads/2019/10/3.Syrian-Children.CaseStudy.pdf). This initiative is closely aligned with ACEV's Preschool Education Program (PEP), implemented since 2003 in areas in Turkey where access to early childhood education rates is low [74,75]. As in other regions of the world, the COVID-19 pandemic and associated lockdowns has posed a grave challenge to preschool educational opportunities [76].

**Jordan.** A number of organizations are refining ECD programs that work directly with families to help alleviate profound stress and build prosocial behavior and resilience. In Jordan, a consortium of local and international scholars worked with Mercy Corps and Syrian/Jordanian communities to evaluate a structured psychosocial intervention implemented with refugee and war-affected youth [77,78]. The randomized clinical trial assessing one such program focused on the biological, cognitive, and psychosocial impacts of stress alleviation for 11–18-year-olds, in ways that explicitly built on sustainable and equitable research and policy partnerships [112]. The project was featured in the award-winning short documentary, *Terror and Hope: The Science of Resilience*, to exemplify how science, fueled by compassion, can work to break the impacts of violence - and foster constructive dialogue between youth, families, researchers, practitioners, and policymakers (see: https://www.ronbourkefilms.com/terror-and-hope).

Many programs and interventions have been designed, evaluated and led by people from conflict-affected regions. One noteworthy example is We Love Reading (WLR), an innovative approach developed in Jordan, by Dr. Rana Dajani, to have adult volunteers read aloud to groups of children in their local community (https://welovereading.org/). It is a practical and sustainable grassroots approach to stimulate learning, using storybooks to foster the love of reading. Being volunteer-driven, it empowers people in marginalized communities to become social entrepreneurs and to co-develop books for children. The community-based approach is vital and fostered through interpersonal engagement. This initiative has won multiple international awards for social innovation and refugee education, and has now spread, from a local solution, to as many as 63 countries globally. Its impact on child development outcomes is now being rigorously evaluated [111].

Both examples from Jordan highlight critical components of successful programs, namely strong partnerships, cultural engagement, and structural competency. Strong partnerships work to bring together local, regional, and international actors in ways that foster pathways to health, development, and social inclusion [112,113]. Cultural engagement matters to integrating policies and programs with teaching and parenting practices [118]. Structural competency is needed to understand how race, ethnicity, inequality, and discrimination work themselves into programmatic outcomes at individual and community levels. (see: https://scholarworks.wmich.edu/jssw/vol46/iss4/5/). These aspects of research, policy, and practice synergistically affect the effectiveness and longevity of specific programs; they are cross-cutting determinants of impactful ECD programs in the context of social transformation.

Of course, presenting examples of successful ECD programs is only a first step. We do not make an argument that the primary responsibility for positive development and larger societal outcomes is at the individual-level, given that children are embedded within social, economic, and political structures [120]. On the contrary, addressing social, economic, and political drivers of conflict and injustice is essential to promote positive child development and sustainable societal change.

## 6 Knowledge transfer and social action

Evidence-based recommendations, regarding how best to strengthen national policies and programs as well as frame the holistic needs of the developing child, have been articulated in The Lancet ECD series [5]. In what follows, we comment on the four action items that we need to take in light of the realities of today's world in order to provide concrete solutions. The problems of ECD practice in crisis- and low-resource settings can only be effectively addressed by long-term, mutually beneficial, and equitable collaborations between researchers, practitioners, and communities who evaluate and apply such programs. Thus, action plans are now inviting individuals in academia and applied ECD settings to create a joint vision on how best to support young children and their families around the globe. The aforementioned networks (e.g., ECPC, ECDAN) are formidable examples of collaborative efforts that directly translate research into policy and practice.

**Action item 1: *Expand political will and*** funding ***through advocacy for ECD-related SDGs****.* Under the SDG umbrella, investing in ECD is not only an aim in itself, but it is also a requisite for achieving other SDGs. For example, SDG 4.2 calls for universal access to quality ECD, care, and pre-primary education, and provides unprecedented opportunity to scale-up ECD services. Convincing government leaders and policymakers to take action at present, during the ongoing pandemic and the accompanying political and economic challenges, is an ambitious goal. That said, the data concerning the long-term value of delivering high quality ECD services are compelling [79,80]. An effective strategy is in-person advocacy, e.g., meeting with government officials to share personal stories of families that have benefited from high-quality ECD services.

There is certainly a need to foster strong, sustained engagement with local communities. One way is to co-produce and disseminate documents in local languages in order to convey scientific findings from global ECD programs. Developing a compelling Theory of Change is another step in the right direction (see: https://ecdpeace.org/work-content/theory-change-early-childhood-development-sustainable-development). Engaging the media, as with the documentary *Terror and Hope: The Science of Resilience*, is another important opportunity to reach wider audiences, in ways that can convince citizens and policymakers that science-based, high-quality programs are worth the investment. In addition, in order to ensure sustainability and scalability, partnerships and communication mechanisms to link civil society and social entrepreneurs are critical. Applied research studies have increasingly explored advocacy strategies to influence early childhood education systems, for example in countries like Jordan [117] and Rwanda [121].

**Action item 2: *Create a policy environment that*** supports ***nurturing care.*** Efforts to improve early childhood development and ensure that all children receive the five components of nurturing care require the support of government officials and policymakers who need to identify gaps, set priority areas for intervention, and develop sustainable and cost-effective action plans [81,82]. For example, the transformative policies recommended by UNICEF to support children during the COVID-19 pandemic include: ensure all children learn by closing the digital divide; guarantee access to health and nutrition services, and make vaccines affordable and available; support and protect mental health, and take steps to bring an end to abuse, gender-based violence, and neglect in childhood; increase access to clean water, sanitation and take steps to address the impact of climate change; reverse the rise in child poverty and ensure an inclusive recovery for all by providing paid parental leave; and mitigate the vulnerability imposed by the COVID-19 pandemic on migrant, displaced, and refugee children, as well as those living in crisis-affected countries. Consequently, there is a clear need to redouble efforts to protect and support children and their families living through conflict, disaster, and displacement especially during the ongoing pandemic [83,84].

**Action item 3: *Build capacity to promote ECD through multi-sectoral coordination.*** In each country and community setting, interventions that can be implemented by both governments and NGOs, are highly dependent on the availability of skilled human resources to provide equitable access. The best way to reach large numbers of families and children involves the integration of ECD interventions into existing service delivery platforms such as health and nutrition services, starting with family planning and routine antenatal care, and continuing through early childhood [85]. The enabling environment created through these policies will serve to support parents and elevate the importance of parenting as an accelerator to achieve optimal developmental outcomes for the next generation. The COVID-19 pandemic spurred a generation of new educational materials for responsive and positive parenting. Through a collaboration between UNICEF, WHO, CDC and major NGOs, these evidence-based tools were made available online, through radio broadcasts, rapid short message service (SMS), and in-country programs to more than 150 million families in 60 countries (see: https://www.savethechildren.org/content/dam/usa/reports/ed-cp/save-our-education-report.pdf; https://www.covid19parenting.com/digitalparenting).

In the context of the ongoing pandemic, ECD practitioners and scholars have launched interactive web-based services for parents that have been widely disseminated (see: https://www.covid19parenting.com/home). Formats include tip sheets, comic strips, radio sketches, TV broadcasts, public service announcements via loudspeakers and community radio, community worker templates, webinars, social media messaging, interactive text messages, online parent support groups, phone-based counselling, and theme songs. Parenting intervention content can also be delivered through mobile applications, texting, and chat services (see: https://www.covid19parenting.com/digitalparenting). Comparable efforts are underway around the world including in the Middle East and North Africa (MENA) region, thanks to the efforts of the Arab Resource Collective (ARC) (see: https://mawared.org/en/).

**Action item 4: *Ensure accountability for ECD services, increase research, and foster global and regional leadership and action.*** Ensuring the inclusion of a core set of ECD indicators is of paramount importance. Research that links detailed longitudinal data on policies and programs with outcomes, allowing causal modelling, is essential to ensure continuing progress of the field [86]. The recently released “Early Childhood Development Index” ECDI-2030 (see: https://indicators.report/indicators/i-32/) has been endorsed by the United Nations Statistical commission as the tool to measure SDG 4.2.1 and is the first ever population-based tool for measuring young children's development. A continued challenge of the field includes the harmonization of global measurement frameworks and the extent to which these are contextualized across geographies. It is imperative to include the participation of local communities into how those frameworks are formulated, implemented, and ultimately applied to strengthen service provision and guarantee the rights of children. One of these efforts emerged in response to the need to measure the progress of Target 4.2 of the SDGs (see: http://www.ecdmeasure.org/what-is-melqo/). Global multi sectoral and inter-disciplinary partnerships have operationalized the adaptation and application of these frameworks in Colombia, among other countries [87]. It is imperative to continue to strengthen the ECD measurement agenda while guided by principles of equity, local agency and participation, and do-no-harm.

## 7 How can youth become agents of change?

We clearly need to support the next generation of youth as partners in decision-making and in the peacebuilding processes, as stipulated in the United Nations resolution (UNSCR 2205) on Youth, Peace and Security, adopted by the United Nations Security Council in 2015 [88]. The world is home to 1.8 billion adolescents, and young people constitute the human capital of most low- and middle-income countries (LMICs). Adolescents can provide novel and fresh solutions to peacebuilding through ECD, and their involvement in ECD services represents an opportunity to address the workforce gap [89]. Young people's meaningful involvement in peacebuilding initiatives addresses their aspirations and enhances their political awareness [90]. Since adolescence is a period of significant identity and moral development [91], such involvement might inspire many of them to dedicate their lives to advance peacebuilding and ECD services, promoting their full and effective participation [89,92].

The current reality, however, is that young people are still largely sidelined. Young people, especially young girls, in many regions of the world continue to be socially, economically, and politically excluded. The recent WHO–UNICEF–Lancet Commission identified several barriers to their engagement including traditional hierarchical governance and adultism [7]. Political polarization and instability, armed conflicts, forced migration, climate change and the ongoing COVID-19 pandemic have further increased the vulnerabilities of many young people. These realities have led to the formation of a Youth Leadership Initiative within the ECPC, focused on how best to define and implement specific activities and opportunities for youth leaders in alignment with the ECPC's Mission and Theory of Change. Three examples of ongoing projects that engage youth leaders are presented below.

**Pakistan.** In 2015, colleagues at The Edward Zigler Center in Child Development and Social Policy at Yale University partnered with researchers from Harvard University and Aga Khan Universities to develop, implement, and scale-up one of the few robustly evaluated youth-led ECD programs in LMICs. The program is entitled, Youth Leaders for Early Childhood Assuring Children are Prepared for School (LEAPS). LEAPS is a set of cross-generational programs that aim to train, mentor, and empower youth to promote the demand for quality ECD programming in their communities [[93], [94], [95]]. LEAPS is an evidence-based program that is closely aligned with a number of the UN's 2030 SDGs [7]. Given the success of the LEAPS initiative in Pakistan, work is also now underway to explore the feasibility of implementing LEAPS in target municipalities in Colombia.

**Colombia.** Working with local leaders and academic partners, the LEAPS implementing team is currently in the process of rolling out a study to assess: (a) the governmental support and assessment of governance and institutional arrangements for program sustainability and scale-up; (b) the attributes of youth buy-in for program participation and assessment of the benefits of the program for youth professional development in the Colombian context; (c) the nature of community and family perceptions and level of acceptability of youth-led programming for early childhood; and (d) the processes to establish academic partnerships and mentoring for sustained program implementation and evaluation. The initial plan is to conduct a qualitative study to characterize the above areas, as a first step to determine key attributes of the program's adaptation. The data will then be utilized as a key set of preliminary findings to rollout a culturally adapted and contextualized youth led ECD program in Colombia.

**Middle East.** The Seeds of Peace program focuses on personal transformations of youth leaders to inspire wider societal change. The program begins with a month-long summer camp in the United States in the State of Maine. The campers are adolescents between 14 and 16 years of age who come from all across the globe, especially in areas affected by ongoing conflict including the Middle East. The selection process is competitive, and more than 300 campers are selected each year. For nearly 2 h each day, the campers engage each other directly in small-group dialogue sessions organized by conflict region. Together with their mentors, usually former campers, they tackle the most distressing and divisive issues defining their conflict, sharing their personal experiences, reflecting on competing narratives, and challenging each other's perspectives. Ms. Haifa Staiti, a Seeds of Peace graduate and the founder of Empathy for Peace - an NGO based in Canada (see: https://www.empathy-for-peace.org/) reported that the most transformative aspect of her experience at the Seeds of Peace camp, in the late 1990s, was the opportunity to connect with knowledgeable, nurturing, and empathetic adult counselors who provided support and care during the month-long camp. Counselor support, combined with the opportunity to connect with youth from “*the other side of the conflict*”, helped “*sow the seeds for new ways of thinking, seeing and being in the world that contributed to the person I grew up to become*”. This experience inspired her to establish the Canadian NGO, Empathy for Peace (see: https://www.seedsofpeace.org/).

In considering the role of children and youth in building more peaceful communities, it is essential to reflect on the existing scientific literature concerning the transformative power of empathy, especially empathy for members of the “out group” as well as the impact of ongoing youth-based peacebuilding and conflict transformation programs that have been established around the world [96].

In addition to the U.S.-based Seeds of Peace initiative, there are many similar exemplars operating globally, including the Heart-to-Heart program in Canada (see: https://www.heart-to-heart.ca), and the Whitaker Peace and Development Initiative, which operates in the United States as well as in countries in Africa and South America (see: https://www.wpdi.org). As mentioned before, there are also multiple examples of local initiatives and organizations that support young people's involvement in peacebuilding such as the Africa Youth for Peace and Development (https://africayouth.webs.com) and the Sou da Paz Institute in Brazil (https://soudapaz.org).

In moving forward, the ECPC, Seeds of Peace, Empathy for Peace, and similar organizations will need to continue to develop and sustain their regional and global opportunities of mentorship for future youth leaders. They will continue to explore with the youth what concrete steps can be taken at the present time to enhance their involvement in the global movement to build social cohesion and a Culture of Peace. Ideally, ethnically and geographically diverse working groups of youth representing under-represented populations can be included as co-creators and evaluators of solutions that have been fully informed by their specific needs and priorities. Their voices, too, need to be heard. The scientific community can encourage promising young scholars to further explore and expand our understanding of the biobehavioral systems that underlie love and sociality.

By incorporating ECD strategies, children and youth-based peacebuilding initiatives may further increase the degree of participant self-transformation. These interventions can have a multiplier effect if participating children and youth are also explicitly educated about the importance of ECD, particularly nurturing care, in relation to their human development and capacity to build more peaceful communities. In doing so, children and youth may be inspired to advocate for ECD initiatives in their homes, and in their adulthood, advocate for ECD investments in their communities. They may develop into future leaders who will enforce wider scale ECD policies and programs in their nations. However, advocacy without structural change is limited.

## 8 Conclusion

Evidence from across a number of scientific disciplines clearly demonstrates the lasting beneficial impact of ECD interventions - from pre-gestation onwards - and from generation to generation. But sadly, many children and their families are exposed to environments that significantly limit the amount of nurturing care children receive. Indeed, there is also clear evidence that a great many children from around the globe are being exposed to increased violence online (via the internet and other digital communications) and in their homes, schools, communities, and societies. To break the cycle of violence and promote peaceful societies, there is a clear need for scholars, practitioners, policymakers, and other civic society leaders to partner with children, youth, and families to build more peaceful, gender equitable, socially cohesive, and resilient communities. In building back better, governments and policymakers, supported by the international community, need to prioritize investments in ECD programs and services, including initiatives that address the ongoing pandemic. However, efforts to support ECD must go hand-in-hand with initiatives to ameliorate structural injustices and institutionally entrenched inequalities that deeply impact development, including poverty and racial discrimination. Through strong coalitions and determined efforts to invest in children and the societies they live in, we can build a peaceful, equitable, just, and sustainable world and drive social change leaving no child and family behind.

## Funding source

This research did not receive any specific grant from funding agencies in the public, commercial, or not-for-profit sectors.

## Declaration of competing interest

The authors declare that they have no known competing financial interests or personal relationships that could have appeared to influence the work reported in this paper.
